# Oncofertility in Women with Renal Cell Carcinoma in the Immune Checkpoint Inhibitors Era: A Multidisciplinary Perspective

**DOI:** 10.3390/jcm15062452

**Published:** 2026-03-23

**Authors:** Michele Miscia, Antonio Raffone, Veronica Mollica, Pietro Piazza, Linda Cipriani, Manuela Maletta, Stefano Ferla, Maria Perucci, Federica Cortese, Irene Pesaresi, Enrico Pazzaglia, Luigi Cobellis, Renato Seracchioli, Diego Raimondo

**Affiliations:** 1 Division of Gynaecology and Human Reproduction Physiopathology, IRCCS Azienda Ospedaliero-Universitaria di Bologna, 40138 Bologna, Italy; renato.seracchioli@unibo.it (R.S.); linda.cipriani@aosp.bo.it (L.C.); manuela.maletta2@unibo.it (M.M.); 2 Department of Medical and Surgical Sciences (DIMEC), University of Bologna, 40126 Bologna, Italy; maria.perucci@studio.unibo.it (M.P.); federica.cortese3@studio.unibo.it (F.C.); irene.pesaresi@studio.unibo.it (I.P.); enrico.pazzaglia@studio.unibo.it (E.P.); 3 Department of Woman, Child, and General and Specialized Surgery, University of Campania “Luigi Vanvitelli”, 80138 Naples, Italy; luigi.cobellis@unicampania.it; 4 Division of Medical Oncology, IRCCS Azienda Ospedaliero-Universitaria di Bologna, 40138 Bologna, Italy; veronica.mollica7@gmail.com; 5 Division of Urology, IRCCS Azienda Ospedaliero-Universitaria di Bologna, 40138 Bologna, Italy; pietro.piazza2@unibo.it; 6 Division of Gynecologic Oncology, Humanitas San Pio X, Via Francesco Nava 31, 20159 Milan, Italy

**Keywords:** renal cell carcinoma, immune checkpoint inhibitors, fertility preservation, ovarian reserve, pregnancy, endocrine immune-related adverse events, tyrosine kinase inhibitors, oncofertility, adjuvant therapy, reproductive health

## Abstract

**Background/Objectives**: Renal cell carcinoma (RCC) care has been reshaped by immune checkpoint inhibitors (ICIs), now used across adjuvant and metastatic settings as PD-1/PD-L1 blockade alone, combined with anti-CTLA-4 agents, or in combination with vascular endothelial growth factor (VEGF)-targeting tyrosine kinase inhibitors (TKIs). As survival improves and systemic therapy courses extend, survivorship priorities—including fertility preservation, reproductive endocrine health, contraception, and pregnancy counselling—become increasingly relevant, even though RCC-specific oncofertility evidence remains sparse. This review examines the biological rationale and clinical considerations underpinning reproductive counselling for women of reproductive age exposed to ICIs (alone or with TKIs) in RCC. **Methods**: A narrative review was conducted in accordance with the SANRA framework, integrating targeted PubMed/MEDLINE searches up to 20 February 2026 and consultation of regulatory product labels to synthesize mechanistic, clinical, and safety data relevant to fertility, endocrine function, contraception, pregnancy, and breastfeeding in RCC. **Results**: We delineate the contemporary RCC treatment landscape to identify feasible timepoints for fertility preservation discussions and propose a pragmatic, implementation-oriented counselling framework that distinguishes evidence-secure recommendations (pregnancy avoidance during therapy, endocrine monitoring, agent-specific washout) from extrapolative domains (long-term ovarian reserve effects and post-ICI periconception safety beyond label intervals). **Conclusions**: By integrating a ‘multi-hit’ biological rationale, treatment context, and available human data, this review highlights RCC-specific research priorities while supporting transparent, evidence-aligned, and multidisciplinary counselling for both fertility preservation and pregnancy safety in the ICI era.

## 1. Introduction

Renal Cell Carcinoma (RCC) remains a major global health burden, and fertility preservation is an increasingly important issue in reproductive-age patients [1]. In 2022, the WHO/IARC Global Cancer Observatory estimated 434,840 new kidney cancer cases and 155,953 deaths worldwide, with a marked male predominance overall [2]. In Italy, the corresponding estimate was 13,666 new cases and 4592 deaths in 2022 [3]. Although RCC is classically a disease of older adults, epidemiologic work consistently shows that a clinically meaningful minority of cases arise in young adults, and incidence rates and trends in the 20–39-year age band have been described in dedicated analyses, raising the relevance of fertility and long-term endocrine health in selected patients [4]. Global datasets confirm a non-negligible kidney cancer burden, including in Italy [2,3]. In parallel, improving survival in adolescent and young adult oncology has made fertility and late endocrine effects a core survivorship priority [5].

Over the last decade, RCC management has shifted from vascular endothelial growth factor (VEGF)-targeting tyrosine kinase inhibitors (TKIs) monotherapy to immune-based strategies, including adjuvant PD-1 blockade for selected high-risk resected disease and first-line metastatic regimens combining immune checkpoint inhibitors (ICIs) with another ICI targeting CTLA-4 blockade or VEGF-targeted TKIs [6,7,8,9,10,11,12,13,14,15]. This evolution has improved disease control and, in subsets, enabled durable treatment-free intervals and long-term survival [9,10,11,12,13]. In parallel, the survivorship “time cost” of therapy (months to years of mandatory pregnancy avoidance plus post-treatment contraception intervals) can become clinically relevant for women approaching advanced reproductive age, even when the probability of permanent gonadal failure is uncertain. This reframes oncofertility in RCC: counselling is not only about classical gonadotoxicity, but also about reproductive timing, endocrine immune toxicities, and pregnancy safety [16,17,18,19,20,21,22,23,24].

RCC-specific evidence on fertility outcomes is limited, for predictable reasons (age distribution, low absolute numbers of young women, historical focus on life-prolongation) [6,8,14]. However, three converging factors justify a focused synthesis: (i) expansion of ICIs into potentially curative adjuvant settings with finite treatment duration [10,15], (ii) increasing chronicity of metastatic RCC care with prolonged exposure to ICIs and TKIs [9,11,12,13], and (iii) the recognized spectrum of endocrine immune-related adverse events (irAEs) that can directly impair fecundability and menstrual function if unrecognized [21,22].

This review provides a clinically anchored, multidisciplinary framework for reproductive counselling in women with RCC exposed to ICIs, either alone or combined with TKIs. We integrate epidemiology, mechanism, clinical context, and implementation considerations, and we explicitly separate evidence-secure recommendations from domains where counselling must extrapolate from mixed-tumour ICI literature [17,18,19,20,21,23,24].

## 2. Materials and Methods

This narrative review was structured according to the SANRA framework to improve transparency [25]. PubMed/MEDLINE searches were performed up to 20 February 2026 using free-text terms and field tags covering (i) RCC and disease setting (“renal cell carcinoma”, “RCC”, “advanced”, “metastatic”, “high-risk”, “adjuvant”, “perioperative”); (ii) ICIs and key agents (“immune checkpoint inhibitor”, “PD-1”, “PD-L1”, “CTLA-4”, “nivolumab”, “pembrolizumab”, “ipilimumab”); (iii) VEGF-targeted TKIs commonly combined with ICIs in RCC (“tyrosine kinase inhibitor”, “VEGF”, “axitinib”, “cabozantinib”, “lenvatinib”, “sunitinib”, “pazopanib”); and (iv) reproductive and endocrine outcomes (“fertility preservation”, “oncofertility”, “ovarian reserve”, “AMH”, “pregnancy”, “contraception”, “breastfeeding”, “hypophysitis”, “thyroiditis”). We focused on English-language peer-reviewed literature and consulted regulatory product labels (EMA SmPC; FDA Prescribing Information) for contraception, washout, and lactation recommendations; given the narrative scope, no formal risk-of-bias tool was applied.

Representative executed strings included: (“renal cell carcinoma” OR “RCC”) AND (“immune checkpoint inhibitor” OR “PD-1” OR “PD-L1” OR “CTLA-4” OR nivolumab OR pembrolizumab OR ipilimumab); (“renal cell carcinoma”) AND (“axitinib” OR “cabozantinib” OR “lenvatinib” OR “sunitinib” OR “pazopanib”); (“immune checkpoint inhibitor” OR “PD-1” OR “PD-L1”) AND (“fertility” OR “ovarian reserve” OR “AMH” OR “pregnancy” OR “breastfeeding” OR “contraception” OR “endocrine immune-related adverse events”).

Records were prioritized when they directly addressed RCC treatment context, ICI or ICI–TKI exposure, endocrine irAEs, pregnancy outcomes, or operational fertility preservation pathways. Although no formal risk-of-bias assessment tool or systematic inclusion/exclusion criteria were applied, priority was given to RCC-specific studies, clinical guidelines, and regulatory documents where available, whereas case reports, small case series, and pharmacovigilance data were used more selectively, mainly to capture rare safety signals or clinically relevant scenarios not otherwise represented in larger datasets. Additional references were identified by citation chasing from key guidelines and reviews [6,14,15,20,21,23,24]. Consequently, the conclusions should be interpreted as a conceptual and clinically oriented synthesis of the available evidence rather than a quantitative or systematic evaluation of reproductive risk associated with these therapies.

## 3. Biological Rationale

A mechanistic discussion is necessary because RCC-specific reproductive outcomes remain sparse, and counselling often relies on plausibility plus mixed-tumour clinical data. Three non-mutually exclusive pathways are most relevant: (i) immune-mediated ovarian and endometrial effects related to checkpoint blockade; (ii) VEGF-pathway inhibition disrupting follicular and luteal vascular physiology; and (iii) indirect endocrine dysfunction from irAEs and TKIs affecting thyroid and pituitary axes [21,22,26,27,28,29]. Importantly, these pathways do not map neatly onto the traditional “gonadotoxic vs. non-gonadotoxic” dichotomy used for chemotherapy. With ICIs and TKIs, clinically meaningful reproductive impact may arise from subclinical ovarian inflammation, altered ovulatory/luteal physiology, endocrine derangements, and prolonged pregnancy avoidance, rather than from immediate, dose-dependent follicle ablation [16,21].

Checkpoint pathways are central to peripheral tolerance. PD-1/PD-L1 and CTLA-4 blockade increases autoreactivity and underpins irAEs [21,22]. The female reproductive system is immunologically specialized: ovulation is a controlled inflammatory event; follicle rupture and luteinization involve local cytokine signalling; and corpus luteum formation depends on immune–vascular coordination [21,26]. This implies a narrow tolerance for immune dysregulation. When checkpoints are inhibited, immune activation may extend beyond tumours to normal tissues, including gonadal and endocrine organs, with effects that may be delayed, intermittent, or clinically silent until reproductive testing is performed [21].

Preclinical models support biological plausibility for ovarian injury under checkpoint blockade. Experimental PD-1/CTLA-4 inhibition has been linked to intra-ovarian immune activation, cytokine release (including TNF-α in some models), and follicular loss [16,27,30]. Impaired oocyte competence and reduced oocyte quality have been reported in other murine checkpoint-inhibition models [30]. These observations converge on a concept that is clinically relevant even if effect sizes in humans prove small: checkpoint inhibition can shift the ovarian microenvironment toward inflammatory signalling that is incompatible with follicle survival or oocyte quality in susceptible individuals [16,21,27,30]. Mechanistically, this has been framed as immune-cell infiltration and cytokine-driven apoptosis within the follicular compartment, rather than direct DNA damage [16,27,30]. This matters for counselling because it suggests that any fertility impact, if present, may not manifest as abrupt amenorrhea; instead, it may appear as subtle declines in ovarian reserve markers, altered response to stimulation, or menstrual irregularity during or after exposure [21].

Pregnancy safety is mechanistically distinct from fertility. Maternal–fetal immune tolerance is not passive; it depends on active regulation at the decidua–placenta interface, where trophoblasts and maternal immune cells interact. PD-1/PD-L1 signalling contributes to limiting maternal effector responses against fetal antigens, and CTLA-4 signalling supports regulatory T-cell function [21,26]. Pharmacologic blockade therefore raises coherent concerns for implantation failure, miscarriage, placental dysfunction, fetal growth restriction, and immune-mediated neonatal effects [21,26]. These concerns are reinforced by pharmacology: pembrolizumab and nivolumab are IgG4 antibodies, and transplacental IgG transfer increases later in gestation, implying that fetal exposure could be greater in the second and third trimesters than in early pregnancy [31,32,33,34,35]. Although real-world pregnancy exposures are typically inadvertent and data are heterogeneous, the mechanistic rationale supports the conservative regulatory position that pregnancy should be avoided during exposure and for a defined interval after the last dose [31,32,33,34,36,37,38].

VEGF signalling adds a second, partially independent axis. Ovarian function is highly vascular dependent. Dominant follicle selection, ovulation, and corpus luteum development require rapid angiogenesis and sustained perfusion; luteal progesterone output depends on this vascular scaffold [29]. VEGF inhibition can therefore plausibly induce functional suppression of ovulation and luteal sufficiency and may impair implantation or early pregnancy maintenance through vascular mechanisms [29]. This is not only theoretical: experimental VEGF blockade in non-human primates can trigger functional luteolysis, underscoring how sensitive the luteal compartment is to VEGF-dependent vascular support [29]. In rodents, sunitinib has been associated with impaired ovulation and adverse reproductive outcomes, supporting class-level plausibility for “functional” reproductive disruption during exposure to multi-target TKIs [28]. In RCC, where ICI–TKI combinations are common in metastatic disease, this pathway is clinically important because it may produce more immediate reproductive disruption during therapy, and it layers onto the strict teratogenic contraindication of TKIs in pregnancy [12,13,39,40,41].

In principle, concurrent vascular impairment and immune-mediated ovarian or endocrine perturbation could also interact in a biologically amplifying manner, rather than as merely parallel toxicities. A VEGF-dependent reduction in follicular or luteal vascular support may render the ovarian microenvironment more susceptible to the functional consequences of inflammatory signalling, while immune dysregulation could further compromise tissue homeostasis and recovery. However, although this hypothesis is consistent with the proposed multi-hit framework, direct evidence demonstrating that ICI–TKI combinations cause a more severe or less reversible decline in ovarian reserve than monotherapy or sequential exposure is currently lacking. This possible interaction should therefore be regarded as biologically plausible but unproven, and a priority for future translational and clinical investigation.

Finally, endocrine irAEs represent the most actionable reproductive toxicity domain. Thyroid dysfunction is among the most frequent endocrine irAEs with PD-1/PD-L1 inhibitors, whereas hypophysitis and broader pituitary axis dysfunction are more common with CTLA-4–containing regimens and dual checkpoint blockade [21,22,42]. Even when ovarian reserve is preserved, untreated hypothyroidism can impair ovulation and increase pregnancy complications, and hypogonadotropic hypogonadism from pituitary dysfunction can cause amenorrhea and infertility [21,22]. A systematic review and meta-analysis across checkpoint regimens quantified meaningful incidence of endocrine dysfunction and highlighted regimen-dependence, which is directly relevant to RCC where both PD-1 monotherapy (adjuvant) and dual checkpoint strategies (metastatic) are used [42]. Clinically, endocrine irAEs function as a “multiplier”: they can convert a potentially modest direct gonadal risk into clinically meaningful subfertility if diagnosis and replacement are delayed [21,22].

**Figure 1:**
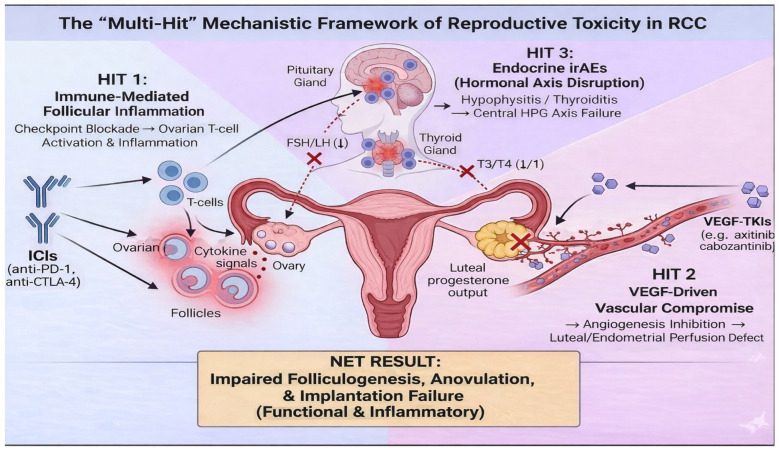
The “multi-hit” mechanistic framework of reproductive toxicity in RCC treated with combination systemic therapy. The schematic illustrates three converging pathways through which immune checkpoint inhibitors (ICIs) and VEGF-targeting tyrosine kinase inhibitors (TKIs) may impair female reproductive function, beyond classical cytotoxicity. (1) Immune Hit (ICI-driven): Checkpoint blockade releases peripheral tolerance, potentially leading to T-cell activation and inflammatory signalling within the ovarian microenvironment, affecting follicular integrity. (2) Vascular Hit (TKI-driven): VEGF inhibition disrupts the intense angiogenesis required for corpus luteum formation and function, impairing progesterone output essential for implantation and pregnancy maintenance (a functional defect). (3) Endocrine Hit (ICI-driven): Immune-related adverse events (irAEs) affecting the pituitary (hypophysitis) or thyroid (thyroiditis) disrupt the hypothalamic-pituitary-gonadal (HPG) axis, leading to anovulation secondary to hormonal dysregulation.

In aggregate, mechanistic evidence supports a multi-hit framework for reproductive risk in RCC: ICIs may contribute to ovarian immune perturbation and endocrine irAEs, while VEGF-targeted TKIs may disrupt the vascular physiology required for ovulation and luteal support and impose strict pregnancy contraindications (Figure 1) [21,22,26,28,29]. This framework does not justify deterministic conclusions about infertility, but it does justify structured, anticipatory counselling and systematic endocrine monitoring in reproductive-age women exposed to these agents [16,21].

## 4. Clinical Context

Oncofertility timing is dictated by treatment intent and regimen structure. RCC management is stage-adapted, spanning surgery and surveillance for localized disease, adjuvant systemic therapy for selected high-risk resected disease, and chronic systemic control for metastatic disease [6,14]. ICIs are embedded across this spectrum as PD-1 blockade alone, dual checkpoint blockade, or ICI–TKI combinations [6,7,8,9,10,11,12,13,14,15]. From a reproductive standpoint, the decisive variables are the time available before systemic exposure, whether therapy is finite or open-ended, and whether TKIs introduce additional reproductive constraints and teratogenic contraindications.

In localized RCC (stages I–II), surgery remains the standard of care, typically partial nephrectomy when feasible, with risk-adapted surveillance thereafter and no routine systemic therapy for low- and intermediate-risk disease [6,14]. In this context, reproductive counselling is usually oriented toward general survivorship and postoperative recovery, and fertility preservation discussions are mainly relevant when additional systemic therapy is anticipated or when disease features and age raise concern for future treatment escalation.

The clearest “curative-intent” immunotherapy scenario is resected high-risk clear-cell RCC, where adjuvant pembrolizumab is a guideline-endorsed option in appropriately selected patients [10,14,15]. In KEYNOTE-564, eligibility encompassed intermediate-high risk and high-risk features as well as patients rendered disease-free after metastasectomy (M1 NED), and randomization occurred within 12 weeks of nephrectomy or metastasectomy with negative margins [15]. Contemporary guidelines align with this definition when recommending adjuvant pembrolizumab [6,14]. This setting is distinctive for oncofertility because treatment duration is finite (one year; 17 cycles of pembrolizumab every three weeks) [15], yet it imposes a predictable interval of mandatory pregnancy avoidance plus post-treatment contraception [31,32,33,34]. For women near advanced reproductive age, this delay can be clinically meaningful even if permanent ovarian failure is unlikely, and it justifies explicit discussion of the “time cost” and the option of fertility preservation before therapy initiation when oncologic timing permits [20,23,24]. The post-surgical interval is often the only predictable pre-treatment window in RCC, and in adjuvant practice it can be leveraged to coordinate referral, complete baseline ovarian reserve assessment if desired, and, when appropriate, perform time-efficient controlled ovarian stimulation using random-start protocols [23,24].

In metastatic RCC, first-line standards are predominantly immune-based combinations guided by prognostic stratification (e.g., IMDC risk categories), which informs expected disease tempo and the tolerance for delaying treatment [6,14]. Dual checkpoint blockade (nivolumab plus ipilimumab) is an established option and can yield durable responses in a subset of patients [9,11]. In CheckMate 214, treatment continued until progression or unacceptable toxicity, reflecting a real-world pattern in which systemic therapy timing is uncertain and pregnancy planning cannot be scheduled prospectively [9]. For counselling, this matters because the feasibility of fertility preservation before treatment is often constrained, and the most realistic near-term goals become effective contraception, documentation of reproductive intent, and proactive endocrine monitoring in anticipation of potential irAEs that may affect menstrual function and fecundability [21,22].

ICI–TKI combinations add a distinct layer of complexity. KEYNOTE-426 (pembrolizumab–axitinib), CLEAR (pembrolizumab-lenvatinib) and CheckMate 9ER (nivolumab–cabozantinib) established immune–VEGF combinations as first-line standards, with ICI exposure often capped in trial designs (e.g., pembrolizumab given for a fixed maximum number of cycles; nivolumab not exceeding two years in CheckMate 9ER), while TKIs can continue until progression or toxicity [12,13,43]. This structure has two implications for oncofertility. First, pregnancy is contraindicated not only during ICI exposure but also during ongoing TKI therapy, and washout intervals are agent specific, making “generic” contraception advice unsafe [31,32,33,34,39,40,41]. Second, even when durable disease control occurs, the presence of a continuously administered TKI can delay any potential treatment-free interval suitable for pregnancy attempts [12,13]. As a result, the metastatic ICI–TKI setting disproportionately emphasizes early contraception planning, explicit counselling about the likely duration and uncertainty of systemic therapy, and rapid coordination with reproductive endocrinology services when a short, clinically acceptable delay for fertility preservation is being considered [20,21,23,24].

Across metastatic strategies, exceptional responders and long-term survivors are increasingly recognized, which makes future parenthood a realistic consideration for a minority of patients [9,11,12,13]. However, the feasibility of pregnancy planning depends on durable remission or stable disease, treatment cessation, endocrine stability, and often on fertility preservation performed earlier when feasible [21]. A pragmatic clinical posture is therefore to treat reproductive counselling as time-sensitive at diagnosis rather than as a survivorship “add-on,” because the opportunity to act—particularly for oocyte or embryo cryopreservation—often exists only before systemic therapy is initiated [23,24]. Evidence from oncofertility implementation research and structured frameworks developed in ICI-treated young women in other tumours emphasizes that early referral and documentation improve decisional quality and reduce regret, even when biological risk is uncertain and counselling must be explicit about evidentiary limitations [16,44].

An RCC-specific nuance is that diagnosis at young age can raise suspicion for hereditary predisposition syndromes [45]. While detailed genetics is beyond this review, clinicians should recognize that genetic counselling/testing may be appropriate in selected patients and that, when a pathogenic variant is identified, fertility preservation can intersect with reproductive planning via PGT-M [46]. From a practical standpoint, PGT-M is embedded in an IVF pathway and ultimately requires embryo testing to identify embryos not carrying the familial pathogenic variant [46]. This has direct implications for oncofertility counselling. It can orient discussions toward embryo cryopreservation when feasible, because embryos constitute the immediate substrate for PGT-M; conversely, when oocyte cryopreservation is chosen (e.g., because embryo creation is not possible or not desired at that time), PGT-M may remain actionable later but will depend on subsequent access to sperm and embryo creation under the applicable regulatory framework [46]. In addition, PGT-M entails a defined pre-analytical and analytical workflow—including genetic counselling, confirmation and classification of the familial variant, and development/validation of a family-specific testing strategy—which can introduce additional coordination requirements and is best anticipated early in the oncofertility pathway [46]. This can influence the preferred fertility preservation strategy and downstream timelines, and it is therefore best addressed early, parallel to systemic treatment planning when clinically indicated [46].

Taken together, the RCC treatment map defines distinct oncofertility scenarios: localized disease usually allows elective counselling; adjuvant therapy provides a predictable post-surgical pre-treatment window and finite exposure; and metastatic disease often mandates urgent therapy initiation with uncertain duration, making early counselling, contraception planning, and expedited referral pathways critical even when fertility preservation itself is not feasible [6,14,15,20,21,23,24].

## 5. Discussion

The most defensible counselling posture in RCC is neither reassurance nor alarm, but structured transparency. Evidence is robust that pregnancy is contraindicated during ICI and TKI treatment; evidence is robust that endocrine irAEs occur at clinically meaningful rates and can impair reproductive function; evidence is weak regarding long-term ovarian reserve trajectories after ICIs, particularly in RCC and in ICI–TKI combinations [12,13,21,22,31,32,33,34,42,47,48,49]. In addition, much of the biological and clinical evidence informing reproductive counselling in this setting derives from mixed-tumour populations, regulatory data, or preclinical studies rather than from RCC-specific cohorts. Accordingly, several considerations discussed in this review should be interpreted as careful extrapolations rather than direct RCC-specific estimates. In this context, counselling should therefore focus on what can be acted on now: timely fertility preservation referral when appropriate, effective contraception and agent-specific washout, endocrine monitoring, and a clear plan for pregnancy attempts only within oncologically safe treatment-free windows. An implementation-oriented counselling framework across adjuvant and metastatic settings is summarised in Table 1.

A recurrent clinical error is treating fertility preservation as relevant only when drugs are “classically gonadotoxic.” In RCC, the principal fertility threat may be time: a year of adjuvant therapy plus washout, or prolonged metastatic therapy with uncertain duration, can push some women beyond a steep age-related decline in fecundity even if ovarian reserve is not directly destroyed by treatment. This logic is increasingly recognized in broader ICI oncofertility work and is directly applicable to RCC [16,20,21,23,24]. In this context, reproductive counselling primarily addresses the protracted delay in childbearing and the uncertain timing of a safe pregnancy window, rather than the absolute risk of iatrogenic sterility. This “time cost” should therefore be addressed explicitly during oncofertility counselling.

Fertility preservation options in RCC should follow standard oncofertility principles and be discussed as early as feasible, ideally before systemic therapy when a pre-treatment window exists [20,23,24,50,51]. In post-pubertal women, the most established cryopreservation strategies are mature oocyte cryopreservation and, where legally permitted and acceptable, embryo cryopreservation, both pursued after controlled ovarian stimulation and oocyte retrieval [23,24]. The choice between oocytes and embryos is primarily driven by partner/donor availability, patient preferences regarding embryo creation, and local regulatory constraints; importantly, both pathways share the same time-critical steps and therefore hinge on whether oncologic scheduling can accommodate a brief delay [23,24]. When helpful for individualized counselling, baseline ovarian reserve assessment (e.g., AMH and antral follicle count) can be documented to contextualize expected oocyte yield and to inform whether one stimulation cycle is likely to be sufficient within a constrained timeline, while remaining explicit that these markers do not guarantee reproductive outcomes [24]. More broadly, age together with baseline AMH and AFC may help contextualize baseline reproductive vulnerability in women who are candidates for ICI- or ICI–TKI-based treatment. Their interpretation can be supported by age-specific reference ranges and percentile-based nomograms, which may offer clinicians a practical framework to identify women with ovarian reserve values lower than expected for their age and therefore potentially more vulnerable to treatment-related delay or dysfunction [52,53,54]. However, these parameters should be regarded as tools for baseline risk contextualization rather than as validated predictors of immune-mediated or permanent gonadal failure.

Contemporary guidance endorses random-start stimulation protocols that can often complete stimulation within roughly two weeks, supporting integration of fertility preservation into time-sensitive cancer pathways [23,24]. Alternative stimulation approaches aimed at attenuating estrogen exposure, such as letrozole co-administration, may be considered on a case-by-case basis when minimization of estradiol peaks is deemed clinically desirable, although a specific evidence base for routine use in RCC is lacking [55]. RCC is not typically managed as a hormone-driven malignancy in the way some breast cancers are, but direct evidence on the safety of transient supraphysiologic estradiol in RCC remains limited. Some translational studies suggest that estrogen-related signalling may influence RCC biology, including in clear-cell disease [56,57,58]. However, no clinical evidence currently demonstrates that specific histological subtypes or metastatic burden confer greater susceptibility to the brief hormonal surges associated with fertility-preservation stimulation protocols. These disease features may nevertheless influence the urgency of systemic treatment and therefore the practical feasibility and acceptable timing of ovarian stimulation. Decisions about stimulation should therefore remain individualized within multidisciplinary discussion, particularly in clinically aggressive disease requiring urgent therapy [6,14,24].

Ovarian tissue cryopreservation (OTC) should be presented as a valid fertility preservation strategy whenever it is feasible and considered safe, including scenarios in which it is preferred by the patient, when ovarian stimulation is undesirable, or when maximizing the range of future reproductive options is a priority [24,59]. OTC entails laparoscopic harvesting and cryostorage of ovarian cortex, with the potential for later autotransplantation to restore endocrine function and, in some cases, fertility; as with other surgical FP procedures, counselling should address invasiveness, center expertise, and the oncologic risk profile, including the theoretical concern of malignant contamination in solid tumours [24,59]. Ovarian metastasis from RCC is rare but documented, and this should be acknowledged whenever OTC is contemplated, with case-by-case oncologic input and careful tissue assessment strategies [24,60]. In vitro maturation may be considered in highly selected settings, but it remains less established than oocyte/embryo cryopreservation and is not RCC specific [24].

Contraception and washout counselling should be explicit and agent specific, because this is one of the few domains where evidence is operationally clear. Regulatory product information contraindicates pregnancy during pembrolizumab or nivolumab exposure and recommends effective contraception during therapy and for a defined interval after the last dose [31,32,33,34]. In practice, this often means at least 4 months after pembrolizumab and 5 months after nivolumab; however, recommendations regarding contraception, washout intervals, and breastfeeding may vary across regulatory jurisdictions (e.g., European Medicines Agency vs. U.S. Food and Drug Administration) and may be updated as new pharmacovigilance data emerge. Therefore, clinicians should verify the most current product label or prescribing information for each specific agent when counselling patients [31,32,33,34]. For VEGF-targeted TKIs, pregnancy is also contraindicated and contraception intervals vary by agent; for example, axitinib requires contraception during therapy and up to 1 week after stopping, whereas cabozantinib requires contraception during therapy and for at least 4 months after stopping, and lenvatinib requires contraception during therapy and for 30 days after the last dose [39,40,41]. Breastfeeding is likewise discouraged during therapy; post-treatment intervals should follow current product labels, and a concise, label-informed summary for selected agents is provided in Table 2 [16,31,32,33,34,39,40,41,61]. This heterogeneity is precisely why “generic washout advice” is unsafe; a concise, documented plan should be part of the baseline counselling record.

Pregnancy after ICIs remains the most emotionally salient question for many patients and clinicians. Mechanistic concerns are strong because PD-1/PD-L1 signalling contributes to maternal–fetal tolerance [21,26]. Clinical data on pregnancy following exposure to ICIs remain heterogeneous and are largely derived from case reports, small case series, and pharmacovigilance databases. Reported outcomes include miscarriages, prematurity, fetal growth restriction, and occasional immune-mediated neonatal complications, but interpretation is constrained by reporting bias, confounding, and missing denominator data [36,37,38]. This heterogeneity limits the ability to establish precise risk estimates and underscores the need for prospective studies and dedicated pregnancy registries for patients exposed to immunotherapy. A recent cohort analysis did not show clear overreporting of adverse outcomes relative to reference groups, but it emphasized limitations and the need for caution [37]. In RCC, these uncertainties are compounded by frequent co-exposure to TKIs, which are teratogenic and contraindicated in pregnancy [12,13,39,40,41]. Therefore, the clinically responsible message is that pregnancy should be avoided during ICI/TKI therapy and deferred until therapy completion plus label-specified washout; any contemplated pregnancy should occur only within a stable oncologic remission/control window and should involve high-risk obstetric expertise [14,31,32,33,34,37,62].

Endocrine irAEs are the reproductive toxicity domain with the greatest immediate clinical leverage. Thyroid dysfunction and hypophysitis are well-described, and incidence varies by regimen, with higher risk in CTLA-4–containing strategies [21,22,42]. For women of reproductive age, baseline endocrine assessment and structured follow-up are practical, low-cost interventions that can prevent prolonged amenorrhea or anovulation and can optimize preconception safety when pregnancy is later pursued [21,22]. In practice, this means proactive screening for symptoms, periodic thyroid function testing, and a low threshold to evaluate pituitary axes when menstrual disruption, fatigue, hypotension, or other suggestive features occur. The goal is not to medicalize all survivorship, but to avoid missed diagnoses that can be corrected and that meaningfully affect reproductive plans [21,22,42].

Operational delivery is often the limiting step. Referral rates for fertility preservation remain suboptimal across oncology, even when guidelines recommend routine discussion and referral pathways [44]. RCC adds complexity because the “risk” is frequently framed as uncertain. Effective implementation of fertility preservation strategies in patients with RCC requires a multidisciplinary approach involving medical oncology, reproductive endocrinology, clinical genetics, and high-risk obstetrics. Early coordination among these specialties can facilitate timely decision-making and help minimize unnecessary delays in oncologic treatment. A workable solution is to define simple triggers that prompt counselling rather than relying on subjective impressions: reproductive age, planned adjuvant therapy with a defined duration, or metastatic therapy expected to be prolonged are reasonable triggers for early counselling and documentation. This aligns with broader ICI oncofertility reasoning: patients value early, honest discussion even when evidence is incomplete, and decisional regret can be reduced when options are presented before time windows close [16,20,23,24].

Finally, research priorities in RCC should be concrete. There is a clear need for prospective cohorts that longitudinally evaluate ovarian reserve markers (e.g., AMH and antral follicle count), menstrual function, endocrine immune-related adverse events, and long-term reproductive outcomes in women treated with ICIs and ICI–TKI combinations in RCC. Such studies would help move the field from mechanistic inference toward more precise clinical risk estimation. Such registries should also incorporate systematic sex-stratified analyses of oncologic and reproductive outcomes, particularly given emerging evidence that sex may influence prognosis under ICI-based therapy in advanced RCC [63]. These efforts should also determine whether combining baseline ovarian reserve assessment with more mechanistically oriented immune-endocrine biomarkers—such as exploratory anti-ovarian autoantibodies, cytokine profiles, or peripheral immune phenotyping—may improve identification of women at higher risk of clinically meaningful reproductive dysfunction during or after ICI- or ICI–TKI-based treatment. Mixed-tumour pregnancy registries should be expanded with RCC-specific annotation, including ICI-only versus ICI–TKI exposure and precise drug-free intervals prior to conception [21,36,37,38]. Mechanistic work should interrogate whether immune-mediated ovarian injury signals translate into clinically meaningful reserve decline in humans, and whether endocrine irAEs are the dominant driver of reproductive dysfunction in real-world settings [21,30,42]. Until such data exist, RCC counselling must remain transparent about uncertainty while rigorously applying what is already known.

## 6. Conclusions

Oncofertility in RCC is becoming clinically relevant because immune-based regimens are increasingly used in curative-intent adjuvant settings and because metastatic RCC care is more often chronic, producing survivorship horizons in which future parenthood may be contemplated. RCC-specific fertility outcome data are limited, but a practical counselling framework is still possible. The evidence-secure pillars are clear: pregnancy is contraindicated during ICI/TKI therapy; contraception and washout must be agent specific; endocrine irAEs are common enough to merit proactive monitoring; and fertility preservation referral should be offered early when treatment timing and patient goals make it meaningful. The largest uncertainties—long-term ovarian reserve trajectories after ICIs, the incremental effect of ICI–TKI combinations, and post-ICI periconception safety beyond label intervals—should be explicitly communicated as such and prioritized for RCC-specific prospective research. This is particularly important because much of the currently available evidence remains indirect and only partially transferable to RCC-specific counselling. Until these gaps close, high-quality care depends on early counselling, rigorous documentation, multidisciplinary coordination, and a transparent distinction between established evidence and reasonable extrapolation.

## Figures and Tables

**Table 1:** Operational counselling framework in RCC (adjuvant vs. metastatic; ICI-only vs. ICI–TKI).

Setting/Regimen Pattern	Practical Counselling Focus	Fertility Preservation Feasibility	Pregnancy Avoidance & Washout (Principle)	Monitoring Priorities
Resected intermediate-high/high-risk RCC→adjuvant pembrolizumab [10,15]	“Time cost” of finite therapy vs. age-related decline; preserve options before predictable delay.	Often feasible if discussed early post-nephrectomy; random-start stimulation can limit delay [23,24].	Avoid pregnancy during ICI and until label-specified interval after last dose [31,32,33,34].	Endocrine symptoms and thyroid function; document contraception plan [21,22].
Metastatic RCC→dual ICI (nivolumab + ipilimumab→nivolumab maintenance) [9,11]	Contraception, endocrine risk, and realistic future pregnancy planning only in durable responders.	Usually limited; consider only if short delay is oncologically acceptable [23,24].	Avoid pregnancy during therapy; follow label intervals after stopping [31,32,33,34].	Higher endocrine irAE risk; low threshold for pituitary evaluation [21,22,42].
Metastatic RCC→ICI–TKI combinations [12,13]	Strict pregnancy avoidance during dual exposure; plan pregnancy only in stable off-therapy windows.	Often constrained by urgency and continuous therapy; early referral if any delay acceptable [23,24].	Avoid pregnancy during ICI and TKI; washout is agent specific (TKIs vary) [31,32,33,34,39,40,41].	Endocrine irAEs plus vascular/ovulatory effects; coordinate oncology–REI early [21,28,29].
Selected young-age RCC with hereditary suspicion/confirmation	Integrate genetics with reproductive planning where relevant.	FP as appropriate; discuss embryo banking if PGT-M is considered [46].	Avoid pregnancy during ICI and TKI; washout is agent specific (TKIs vary) [31,32,33,34,39,40,41].	Consider genetics referral where indicated; align ART planning with oncology [46].

**Table 2:** Selected ICIs and VEGF-targeted TKIs used in RCC: label-informed contraception, post-treatment interval, and breastfeeding recommendations.

Agent	Pregnancy Avoidance/Contraception During Therapy	Post-Treatment Contraception Interval (After Last Dose)	Breastfeeding Recommendation
Pembrolizumab (PD-1)	Avoid pregnancy; effective contraception during treatment.	4 months	Do not breastfeed during treatment and for 4 months after last dose.
Nivolumab (PD-1)	Avoid pregnancy; effective contraception during treatment.	5 months	Do not breastfeed during treatment and for 5 months after last dose.
Ipilimumab (CTLA-4)	Avoid pregnancy; effective contraception during treatment.	3 months	Do not breastfeed during treatment and for 3 months after last dose.
Axitinib (VEGFR TKI)	Avoid pregnancy; effective contraception during treatment.	1 week	Do not breastfeed during treatment (post-treatment interval not specified in EMA SmPC).
Cabozantinib (multi-target TKI)	Avoid pregnancy; effective contraception during treatment.	4 months	Do not breastfeed during treatment and for at least 4 months after last dose.
Lenvatinib (multi-target TKI)	Avoid pregnancy; effective contraception during treatment.	1 month	Do not breastfeed during treatment and for at least 1 week after last dose.

Note: Recommendations reflect typical regulatory product labels (SmPC/Prescribing Information). Where post-treatment breastfeeding intervals are explicitly stated, they are reported; some labels (including certain EMA SmPCs) may not specify a post-treatment interval. In combination regimens, apply the longest relevant interval among agents. Clinicians should verify the jurisdiction-specific label for each agent and document the agent-specific plan in the counselling record. Sources: regulatory product labels [31,32,33,34,39,40,41,61].

## Data Availability

No new data were created or analyzed in this study. Data sharing is not applicable to this article.

## Abbreviations

The following abbreviations are used in this manuscript:

AFC	Antral Follicle Count
AMH	Anti-Müllerian Hormone
ART	Assisted Reproductive Technology
CTLA-4	Cytotoxic T-Lymphocyte-Associated protein 4
EMA	European Medicines Agency
FDA	Food and Drug Administration
FP	Fertility Preservation
HPG	Hypothalamic-Pituitary-Gonadal
IARC	International Agency for Research on Cancer
ICI	Immune Checkpoint Inhibitor
IgG	Immunoglobulin G
IMDC	International Metastatic RCC Database Consortium
irAE	Immune-Related Adverse Event
M1 NED	Metastatic disease with No Evidence of Disease
OTC	Ovarian Tissue Cryopreservation
PD-1	Programmed Cell Death Protein 1
PD-L1	Programmed Death-Ligand 1
PGT-M	Preimplantation Genetic Testing for Monogenic conditions
RCC	Renal Cell Carcinoma
REI	Reproductive Endocrinology and Infertility
SmPC	Summary of Product Characteristics
TKI	Tyrosine Kinase Inhibitor
TNF-α	Tumour Necrosis Factor alpha
VEGF	Vascular Endothelial Growth Factor
VEGFR	Vascular Endothelial Growth Factor Receptor
WHO	World Health Organization
